# Pharmacoepigenetics: from epigenetic regulation to personalized therapeutics

**DOI:** 10.3389/fphar.2026.1965981

**Published:** 2026-10-16

**Authors:** Ashley M. Nguyen, Tamer E. Fandy

**Affiliations:** Paul L. Foster School of Medicine, Texas Tech University, El Paso, TX, United States

**Keywords:** DNA methylation inhibitors, epigenetics, EZH2 inhibitors, histone deacetylase inhibitors, IDH mutant inhibitors, menin inhibitors, pharmacoepigenetics

## Abstract

Pharmacoepigenetics is an emerging field of precision medicine that examines how epigenetic modifications influence interindividual variability in drug response. This review discusses the clinical utility of epigenetic therapy in personalized medicine. Current FDA-approved epigenetic drugs including DNA methyltransferase inhibitors, histone deacetylase inhibitors, EZH2 inhibitors, mutant IDH inhibitors, and menin inhibitors are reviewed along with emerging therapies such as BET inhibitors, LSD1 inhibitors, precision epigenetic editing, and combination treatment strategies. Despite notable advances, challenges such as target selectivity, dose-limiting toxicity, epigenetic variability and lack of biomarkers for predicting response or selecting patients continue to limit broader clinical implementation of epigenetic drugs. Continued progress is expected to expand the role of pharmacoepigenetics and improve treatment across a growing range of diseases other than oncology.

## 1 Introduction

Pharmacogenomics is the study of how genetic variation across the genome influences drug response, efficacy, and toxicity (Kabbani et al., 2023). It is an essential tool in personalized medicine to improve outcomes and minimize adverse effects from certain therapies (Qahwaji et al., 2024). Epigenetics refers to changes in gene expression executed via mechanisms such as DNA methylation, histone modifications, chromatin remodeling, and non-coding RNA regulation (Dai et al., 2024; Sigalotti et al., 2007). Epigenetic modifications are reversible, do not change DNA sequence and can be influenced by age, lifestyle, environmental exposures, inflammation, diet, smoking, and the microbiome (Celik-Uzuner et al., 2026). Pharmacoepigenetics examines how such epigenetic variability modifies pharmacokinetics, pharmacodynamics, efficacy, toxicity, and resistance to drugs (Smith et al., 2023). Epigenetic regulation of genes involved in drug absorption, distribution, metabolism, and excretion, including cytochrome P450 enzymes and membrane transporters, can alter drug exposure, while epigenetic regulation of drug targets and disease pathways can alter pharmacodynamic sensitivity (Peng and Zhong, 2015; Kacevska et al., 2012; Jin and Zhong, 2023). Consequently, patients with similar genotypes may still differ in treatment response, and an individual’s response may change over time as the epigenome changes. This dynamic layer of regulation distinguishes pharmacoepigenetics from conventional pharmacogenomics and creates opportunities for biomarkers that predict efficacy, toxicity, and resistance.

Epigenetic drugs are therapeutic agents that modulate epigenetic regulators or epigenetic states and thereby alter gene expression. In oncology, established approaches include inhibition of DNA methyltransferases (DNMTs), histone deacetylases (HDACs), EZH2, mutant IDH enzymes, and menin-dependent transcriptional programs (Smith et al., 2023; Ocana-Paredes et al., 2024). Despite the fact that epigenetic therapy was developed for cancer drug discovery, its indications is not restricted to oncology only. Givinostat, an HDAC inhibitor, was approved by the FDA in 2024 for Duchenne muscular dystrophy in patients 6 years of age and older (Xu et al., 2026). Valproic acid also has HDAC-inhibitory activity; however, its established neurologic and psychiatric indications were not originally developed or approved based on its epigenetic profile. Experimental epigenetic interventions are additionally being investigated in kidney disease and neurodegenerative disorders. Preclinical renal studies have implicated DNA methylation, histone modifications, and chromatin regulators in acute kidney injury, diabetic kidney disease, chronic kidney disease, and renal fibrosis, with several epigenetic modulators showing protective effects in experimental models (Ding et al., 2021; Fontecha-Barriuso et al., 2018). In neurodegenerative diseases, HDAC and other chromatin-modifying pathways have been investigated in preclinical models of Alzheimer’s, Parkinson’s, and Huntington’s diseases, although clinical translation remains limited (Xu et al., 2012).

One of the key mechanisms of epigenetic therapy is reactivation of tumor suppressor genes that are often silenced during tumor development and progression (Riyas Mohamed et al., 2025). Epigenetic reactivation of silenced genes has the potential to induce apoptosis or differentiation of tumor cells and increased sensitivity to chemotherapy (Bazinet et al., 2024). Accordingly, epigenetic drugs present a unique approach to cancer treatment with potentially more personalized, precise, and optimized treatment (Ramaiah et al., 2021).

This mini-review discusses the major epigenetic mechanisms that influence therapeutic response; emphasizes how interindividual and intraindividual epigenetic variation can affect drug efficacy, toxicity, metabolism, and resistance. It also reviews currently FDA-approved epigenetic drugs, emerging therapeutic strategies, future directions, and challenges for integrating pharmacoepigenetics into precision medicine.

## 2 Epigenetic biomarkers

Epigenetic biomarkers are measurable features of epigenetic regulation, including DNA methylation, histone modifications, chromatin states, and non-coding RNAs, that can be used for diagnostic, prognostic, predictive, pharmacodynamic, or monitoring purposes (Garcia-Gimenez et al., 2017; Kaminska et al., 2019). In precision therapeutics, predictive biomarkers are particularly important because they can identify patients more likely to benefit from a treatment or experience toxicity, whereas prognostic biomarkers provide information about disease outcome independent of a specific therapy. Effective biomarkers should ideally be specific, sensitive, analytically reproducible, and measurable across clinically accessible specimens (Garcia-Gimenez et al., 2017). Many epigenetic marks are detectable in tissue and circulating nucleic acids, supporting the development of liquid-biopsy approaches.

Epigenetic biomarkers can therefore contribute to disease classification, prediction of therapeutic response, longitudinal monitoring, and patient stratification. O6-methylguanine-DNA methyltransferase (MGMT) promoter methylation is a clinically relevant example of an epigenetic biomarker to predict drug response. MGMT is a DNA repair enzyme that repairs DNA damage induced by chemotherapy. DNA promoter methylation of MGMT suppresses its expression and impairs the repair of temozolomide-induced DNA damage. Consequently, patients with glioblastoma whose tumors harbor MGMT promoter methylation derive greater benefit from temozolomide (Noren et al., 2026), and therefore MGMT methylation status is routinely incorporated into clinical management.

## 3 FDA-approved epigenetic drugs

### 3.1 DNA methyltransferase inhibitors

DNA methylation involves transfer of a methyl group to the C5 position of cytosine to form 5-methylcytosine (Moore et al., 2013). This epigenetic mark can regulate gene expression by recruiting transcriptional repressors and limiting transcription-factor access to DNA. DNMT inhibitors (DNMTi) exert both direct epigenetic and immunomodulatory effects. At pharmacologically relevant lower exposures, the nucleoside analogs azacitidine and decitabine are incorporated into nucleic acids; after incorporation into DNA, they act as suicide substrates that covalently trap DNMT enzymes, leading to DNMT depletion and passive DNA hypomethylation during replication (Figure 1) (Fandy, 2009). At higher doses, DNA damage and cytotoxicity become increasingly prominent. Moreover, DNMTi can also derepress endogenous retroelements, induce viral-mimicry/interferon signaling, enhance antigen presentation, and increase tumor-cell immunogenicity, providing a mechanistic basis for their combinations with immunotherapy (Zhang et al., 2022; Xu et al., 2022). Furthermore, genome-wide and promoter-specific DNA demethylation can reactivate genes involved in differentiation, apoptosis, and immune recognition (Guest et al., 2024).

**FIGURE 1:**
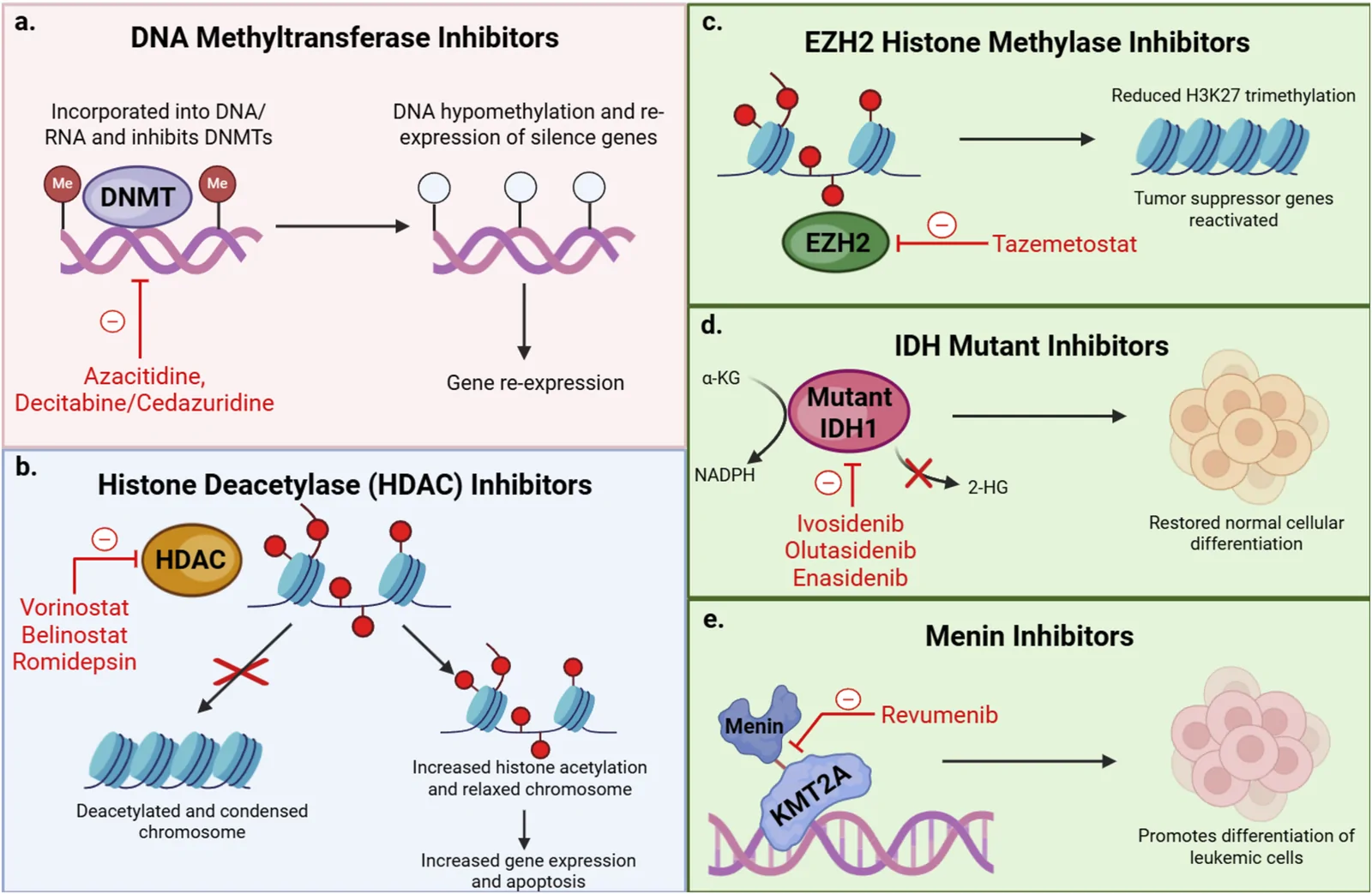
Epigenetic targets and mechanisms of action of FDA-approved epigenetic drugs. **(a)** DNMT inhibitors (azacitidine or decitabine) incorporate into DNA and act as suicide substrates that covalently trap DNMT enzymes, leading to DNMT depletion and passive DNA hypomethylation during replication. **(b)** HDAC inhibitors (vorinostat, belinostat and romidepsin) inhibit HDAC enzymes with consequent increase in histone acetylation (red circles) and relaxed chromatin. **(c)** Tazemetostat is an EZH2 inhibitor that reduces H3K27me3 (red circles) and reactivates gene expression. **(d)** Ivosidenib and olutasidenib reduces 2-HG production by inhibiting mutant IDH1 (enasidenib inhibits mutant IDH2), inducing DNA hypomethylation and restoring cellular differentiation. **(e)** The menin inhibitor revumenib disrupts the binding interface of menin and KMT2A with consequent transcriptional downregulation of HOXA/MEIS target genes and differentiation of leukemic cells. Abbreviations: 2-HG: 2-hdroxyglutarate; a-KG: a-ketoglutarate; DNMT, DNA methyltransferase; EZH2, enhancer of zeste homolog 2; IDH: isocitrate dehydrogenase; KMT2A: lysine methyltransferase 2A; Me: Cytosine methylation.

Azacitidine is an FDA-approved DNMTi used in AML, myelodysplastic syndromes, and juvenile myelomonocytic leukemia (Guest et al., 2024; Jen et al., 2022). Decitabine is another DNMTi that was initially approved by the FDA in 2006 for myelodysplastic syndromes (Kim et al., 2022). Both agents can produce hypomethylation through DNMT trapping and depletion, but their biologic effects are dose- and schedule-dependent, with lower exposures favoring epigenetic reprogramming and higher exposures producing greater cytotoxicity (Riyas Mohamed et al., 2025).

Inqovi is an oral combination of decitabine and cedazuridine approved for adult patients with myelodysplastic syndromes and, in combination with venetoclax, for selected patients with newly diagnosed AML (Kim et al., 2022). Cedazuridine inhibits cytidine deaminase and thereby increases the oral bioavailability of decitabine. The immune effects of DNMT inhibition described above provide an additional rationale for combination approaches, but there are currently no validated predictive biomarkers or companion diagnostics routinely used to select patients for azacitidine, decitabine, or Inqovi on the basis of an epigenetic response signature.

### 3.2 Histone deacetylase inhibitors

Histone deacetylases (HDACs) regulate chromatin structure and transcription through removal of acetyl groups from lysine residues on both histone and non-histone proteins. Deacetylation of histones H2A, H2B, H3, and H4 generally favors chromatin compaction and transcriptional repression (Ramaiah et al., 2021) (Figure 1). HDAC inhibitors differ substantially in isoform selectivity. Vorinostat and belinostat are broad-spectrum or pan-HDAC inhibitors, whereas romidepsin preferentially inhibits class I HDACs, particularly HDAC1, HDAC2, and HDAC3. Increased histone acetylation at promoters of apoptosis- and cell-cycle-related genes can increase transcription and contribute to cell-cycle arrest, differentiation, and apoptosis in tumor cells (Ramaiah et al., 2021).

Vorinostat is a pan-HDAC inhibitor approved by the FDA in 2006 for patients with progressive, persistent, or recurrent cutaneous T-cell lymphoma (CTCL) with cutaneous manifestations (Mann et al., 2007). It binds the catalytic site of HDAC enzymes and increases acetylation of histone and non-histone proteins involved in proliferation, protein stability, motility, apoptosis, and angiogenesis (Maksimova et al., 2023). Belinostat is another pan-HDAC inhibitor approved for relapsed or refractory peripheral T-cell lymphoma and promotes histone acetylation and transcriptional reprogramming (Lee et al., 2015; El Omari et al., 2023). Romidepsin is an FDA-approved Class I-selective HDAC inhibitor for CTCL; after intracellular reduction of its disulfide bond, the active form preferentially inhibits HDAC1/2/3 and increases acetylation of nuclear and cytoplasmic proteins (VanderMolen et al., 2011; Sequera et al., 2025) (Figure 1).

### 3.3 EZH2 inhibitors

Polycomb repressive complex 2 (PRC2) is a multiprotein chromatin-modifying complex whose core components include EZH2 (or EZH1), EED, SUZ12, and RBBP4/7. EZH2 is the catalytic histone methyltransferase subunit and uses S-adenosyl-L-methionine (SAM) to catalyze mono-, di-, and especially trimethylation of histone H3 lysine 27 (H3K27me3). Accumulation of the epigenetic mark H3K27me3 promotes chromatin compaction and transcriptional silencing (Liu and Yang, 2023).

Tazemetostat is an orally active SAM-competitive inhibitor that inhibits wild-type and mutant EZH2, including oncogenic gain-of-function EZH2 variants (Straining and Eighmy, 2022; Gold and Shilatifard, 2024) (Figure 1). It received FDA approval for epithelioid sarcoma and selected follicular lymphoma populations (Hoy, 2020). Tazemetostat was subsequently voluntarily withdrawn from the United States market in 2026 after an increased rate of secondary hematologic malignancies was identified. Currently, no EZH2 inhibitor holds an active FDA approval. Nevertheless, the pathway remains therapeutically active, with next-generation EZH2-selective and dual EZH1/2 inhibitors, including valemetostat, under continued clinical investigation (Keam, 2022).

### 3.4 Mutant IDH inhibitors

Isocitrate dehydrogenase 1 and 2 (IDH1/2) are part of a metabolic family of enzymes that is responsible for the conversion of isocitrate to α-ketoglutarate (α-KG) (Mondesir et al., 2016). Several dioxygenases, including TET2, are dependent on α-KG (Du and Hu, 2021). TET2 is vital to the removal of hypermethylated DNA through the conversion of 5-methylcystosine (5 mC) to 5-hydroxymethylcytosine (5 hmC) and active demethylation. Accordingly, IDH mutations are associated with deficiency of α-KG, accumulation of the oncometabolite 2-hydroxyglutarate (2-HG), lack of TET2 activity, and consequent aberrant DNA hypermethylation and repression of genes vital to cellular differentiation (Du and Hu, 2021).

Ivosidenib is an oral inhibitor of mutant IDH1. In addition to relapsed or refractory IDH1-mutant AML, it is also approved for newly diagnosed IDH1-mutant AML in adults 75 years or older or with comorbidities precluding intensive induction, and for previously treated locally advanced or metastatic IDH1-mutant cholangiocarcinoma (Norsworthy et al., 2019; Li et al., 2026; Woods et al., 2024). Ivosidenib reduces 2-HG production by inhibiting mutant IDH1 and promotes cellular differentiation (Figure 1). Olutasidenib is another oral mutant IDH1 inhibitor approved for relapsed or refractory IDH1-mutant AML and promotes granulocytic and monocytic differentiation (Woods et al., 2025). Enasidenib inhibits mutant IDH2 and is approved for relapsed or refractory IDH2-mutant AML (Stei et al., 2017; Myers et al., 2018). Vorasidenib is a brain-penetrant dual IDH1/IDH2 inhibitor approved by the FDA in 2024 for adult and pediatric patients 12 years and older with Grade 2 astrocytoma or oligodendroglioma harboring a susceptible IDH1 or IDH2 mutation following surgery (Barbato et al., 2025). These approvals illustrate a biomarker-defined therapeutic paradigm in which tumor IDH genotype directly guides drug selection.

### 3.5 Menin inhibitors

Menin is a nuclear scaffold protein encoded by *MEN1* that regulates gene expression through interactions with chromatin-associated protein complexes and transcription factors (Candoni and Coppola, 2024). In KMT2A-rearranged leukemia, menin binds wild-type or chimeric KMT2A-containing complexes and supports aberrant HOXA/MEIS transcription, impaired hematopoietic differentiation, and leukemic transformation (Issa et al., 2023). Revumenib is an oral menin inhibitor that disrupts the menin-KMT2A interface (Figure 1). In NPM1-mutant leukemia, revumenib does not bind mutant NPM1 directly; rather, these leukemias remain dependent on menin-KMT2A activity to maintain HOXA9 and MEIS1 expression, and disruption of this complex promotes differentiation (Issa et al., 2025). Revumenib is FDA-approved for relapsed or refractory acute leukemia with KMT2A translocation and for relapsed or refractory AML with a susceptible NPM1 mutation (Imtiaz et al., 2026; Arellano et al., 2025). This is another example of molecularly defined patient selection for an epigenetic therapy. Table 1 summarizes the indication and adverse effects of FDA-approved epigenetic drugs.

**TABLE 1:** Mechanisms and adverse effects of FDA-approved epigenetic drugs.

Drug	Epigenetic target	Mechanism of action	FDA-approved indication(s)	Major adverse effects/Limitations
Azacitidine	DNMT	Incorporates into DNA/RNA and inhibits DNMTs, resulting in DNA hypomethylation and re-expression of silenced genes	AML, MDS, juvenile myelomonocytic leukemia	Myelosuppression, gastrointestinal toxicity, infection risk
Decitabine/Cedazuridine (Inqovi®)	DNMT	Decitabine inhibits DNMT to promote DNA hypomethylation; cedazuridine inhibits cytidine deaminase to increase oral bioavailability	MDS, AML (selected patients)	Myelosuppression, neutropenia, thrombocytopenia
Vorinostat	HDAC	Inhibits HDAC activity, increasing histone acetylation and expression of genes involved in apoptosis and cell-cycle arrest	CTCL	Fatigue, diarrhea, thrombocytopenia, QT prolongation
Belinostat	HDAC	Pan-HDAC inhibitor that promotes histone acetylation and apoptosis	Relapsed or refractory PTCL	Hematologic toxicity, nausea, fatigue
Romidepsin	HDAC	Selectively inhibits class I HDACs, promoting transcriptional activation and apoptosis	CTCL	Cardiac toxicity, infections, myelosuppression
Tazemetostat^†^	EZH2 histone methyltransferase	Selective inhibitor of wild-type and mutant EZH2, restoring expression of tumor suppressor genes	Epithelioid sarcoma; follicular lymphoma with EZH2 mutation	Fatigue, nausea, cytopenias; safety concerns regarding secondary hematologic malignancies
Ivosidenib	Mutant IDH1	Inhibits mutant IDH1, restoring normal cellular differentiation	Relapsed/refractory IDH1-mutant AML; selected newly diagnosed IDH1-mutant AML; previously treated locally advanced/metastatic IDH1-mutant cholangiocarcinoma	Differentiation syndrome, QT prolongation
Olutasidenib	Mutant IDH1	Selective inhibition of mutant IDH1 promoting myeloid differentiation	Relapsed or refractory AML with IDH1 mutation	Differentiation syndrome, hepatotoxicity
Enasidenib	Mutant IDH2	Inhibits mutant IDH2, restoring normal hematopoietic differentiation	Relapsed or refractory AML with IDH2 mutation	Hyperbilirubinemia, differentiation syndrome
Revumenib	Menin–KMT2A interaction	Blocks menin interaction with KMT2A fusion proteins, promoting differentiation of leukemic cells	Relapsed or refractory AML with KMT2A rearrangement or susceptible NPM1 mutation	QT prolongation, differentiation syndrome, cytopenias
Givinostat	HDAC	Pan-HDAC inhibition increases protein acetylation and modulates pathogenic transcriptional/inflammatory programs	Duchenne muscular dystrophy (patients 6 years and older)	Thrombocytopenia, increased triglycerides, diarrhea, QT prolongation
Vorasidenib	Mutant IDH1/IDH2	Brain-penetrant dual mutant IDH1/2 inhibition decreases 2-HG production	Grade 2 IDH-mutant astrocytoma or oligodendroglioma following surgery	Hepatotoxicity, fatigue, headache, diarrhea

Abbreviations: AML, acute myeloid leukemia; CTCL, cutaneous T-cell lymphoma; DNMT, DNA, methyltransferase; EZH2, enhancer of zeste homolog 2; HDAC, histone deacetylase; IDH, isocitrate dehydrogenase; MDS, myelodysplastic syndrome; PTCL, peripheral T-cell lymphoma.

^†^ The manuscript notes the subsequent U.S. market withdrawal because of safety concerns.

## 4 Emerging therapies and mechanistic trends

### 4.1 BET inhibitors

BET (Bromodomain and Extra-Terminal) proteins, including BRD2, BRD3, BRD4 and BRDT, are a family of epigenetic reader proteins that bind to acetylated histone tails to regulate transcription and the cell cycle (Doroshow et al., 2017). BRD2 binds and activates gene promoters of Cyclin D11, Cyclin E, and several other cell cycle regulatory proteins (Doroshow et al., 2017; Wang et al., 2023). BRD4 interacts with positive transcription elongation factor to initiate and elongate transcription and serves also as a cell cycle regulator. BET proteins are overexpressed in multiple tumor types including hematologic malignancies and solid tumors and drive tumor growth through the activation of proto-oncogenes (Segura et al., 2013). Accordingly, BET proteins are rational targets for cancer therapeutics (Wang et al., 2023). Indeed, BET inhibitors have displayed anticancer activity in multiple types of tumors in preclinical trials. Most BET inhibitors remain in clinical development because of mixed clinical outcomes, dose-limiting toxicities, adverse effects, and the emergence of tumor resistance (Wang et al., 2023).

Pelabresib (CPI-0610) is an investigational pan-BET bromodomain inhibitor that targets the BET family members BRD2, BRD3, BRD4, and BRDT (Rampal et al., 2025). It has demonstrated antiproliferative activity in lymphoma models (Blum et al., 2022). The Phase III MANIFEST-2 trial evaluated pelabresib plus the JAK inhibitor ruxolitinib in JAK-inhibitor-naive myelofibrosis and demonstrated improved clinical responses with a manageable safety profile (Rampal et al., 2025). The regimen also illustrates a modern combination strategy in which transcriptional/chromatin modulation is paired with inhibition of a disease-driving signaling pathway.

### 4.2 LSD1 inhibitors

Lysine-specific demethylase 1 (LSD1/KDM1A) is a flavin adenine dinucleotide (FAD)-dependent amine oxidase that removes mono- and dimethyl groups from H3K4 (H3K4me1/2) and, in specific contexts, H3K9 (H3K9me1/2); because of its oxidative mechanism, it cannot directly demethylate trimethylated lysines (Wang et al., 2026). LSD1 commonly functions in transcriptional repression within CoREST/NuRD-associated complexes and is recruited by SNAG-domain transcription factors such as GFI1 and GFI1B, which is particularly relevant to hematopoietic differentiation (Saleque et al., 2007). LSD1 is overexpressed in multiple cancers and has been associated with adverse prognosis, making it a rational therapeutic target (Wang et al., 2026; Agboyibor et al., 2021).

Iadademstat (ORY-1001) is an investigational, highly selective, irreversible LSD1 inhibitor that covalently modifies the FAD cofactor within LSD1 (Salamero et al., 2020; Maes et al., 2018). In AML models, disruption of LSD1-dependent transcriptional repression, including GFI1/GFI1B-associated programs, promotes monocyte/macrophage differentiation and reduces leukemic stemness (Hartung et al., 2023). Its combination with azacitidine is being evaluated based on complementary epigenetic mechanisms, and clinical development has expanded to regimens incorporating venetoclax (Yu et al., 2026). These combinations are intended to couple differentiation-inducing epigenetic reprogramming with established antileukemic pathways.

### 4.3 Precision epigenetic editing

Conventional CRISPR/Cas9 knockout or knock-in strategies use DNA cleavage followed by repair through non-homologous end joining or homology-directed repair and therefore alter the primary DNA sequence. By contrast, epigenome editing uses programmable DNA-binding platforms, including nuclease-deactivated Cas proteins (dCas), to recruit transcriptional or chromatin-modifying effectors to a defined locus without creating a double-strand break (Nakamura et al., 2021). Early systems fused dCas9 to single effectors such as DNMT3A, TET1, KRAB, or histone-modifying enzymes; however, single catalytic domains can generate incomplete or transient effects. Newer architectures use multivalent or combinatorial recruitment, including DNMT3A-DNMT3L, KRAB-DNMT3A-DNMT3L, SunTag, and related multi-effector systems, to strengthen and stabilize locus-specific chromatin states (Yuan et al., 2026). A clinically important example is CRMA-1001, an investigational epigenetic silencer in a Phase 1/2 study for chronic hepatitis B. CRMA-1001 uses a non-cleaving programmable DNA-targeting system delivered by lipid nanoparticles to direct durable DNA methylation to transcriptionally active episomal and integrated HBV DNA, thereby repressing viral transcription without cutting or changing the HBV DNA sequence (Anglero-Rodriguez et al., 2026). This strategy is particularly attractive in chronic HBV because persistent covalently closed circular DNA (cccDNA) and integrated viral DNA can continue to produce viral antigens even during antiviral therapy.

## 5 Epigenetic combination strategies

Current epigenetic combination strategies has been extended beyond the conventional combination of chemotherapy or radiation with epigenetic modifiers. DNMT inhibitors can derepress endogenous retroviral elements, generate double-stranded RNA, activate type I/III interferon signaling, and increase MHC class I and cancer-testis antigen expression; HDAC and LSD1 inhibition can also remodel antigen presentation and the immunosuppressive tumor microenvironment (Xu et al., 2022). These effects provide a mechanistic rationale for combining epigenetic modifiers with immune checkpoint inhibitors, although clinical benefit has varied by disease and regimen. Another clinically established paradigm is pairing DNA hypomethylating agents with the BCL-2 inhibitor venetoclax in older or medically unfit patients with AML, as an example of coupling epigenetic reprogramming with apoptotic priming. The pelabresib-ruxolitinib regimen in myelofibrosis similarly combines BET-mediated transcriptional modulation with JAK-pathway inhibition (Rampal et al., 2025). Preclinical combinations of HDACi, DNMTi, EZH2 inhibitors, and BET inhibitors with chemotherapy or radiation have also demonstrated radiosensitization, impaired DNA repair, and cell-cycle disruption, but increased toxicity and inconsistent efficacy remain important limitations (Morel et al., 2020). Rational combinations therefore require mechanistic justification, biomarker-guided patient selection, and careful optimization of sequence and dose rather than empiric addition of multiple agents.

## 6 Discussion

Epigenetic profiles change with aging, environmental exposures, disease evolution, and treatment, but the principal translational challenge is not simply epigenetic drift. Precision pharmacoepigenetics requires identification of stable, disease- and tumor-specific predictive signatures that remain informative despite intratumoral heterogeneity and temporal evolution (Celik-Uzuner et al., 2026). Tissue profiling, single-cell epigenomics, and circulating tumor DNA-based methylation assays may help distinguish clinically actionable signals from background variation and enable longitudinal monitoring (Shaman, 2024). These approaches remain technically complex and can be costly, but advances in high-throughput sequencing and liquid-biopsy technologies are improving feasibility.

Although several epigenetic drugs have reached clinical practice, their utility is limited by target selectivity, toxicity, biological context, and resistance. Broad HDAC and DNMT inhibition can alter large portions of the epigenome rather than a single disease-driving locus, and dose-limiting toxicity can restrict treatment intensity. The absence of validated predictive biomarkers for many epigenetic drugs results in empiric treatment based primarily on disease indication. Solid tumors present additional barriers beyond drug penetration and toxicity: relatively low proliferation rates can reduce the activity of S-phase-dependent nucleoside DNMT inhibitors; stromal and microenvironmental barriers can limit drug distribution and immune access; and marked clonal and genetic heterogeneity can provide multiple routes of escape. Resistance mechanisms are also drug-specific. Reduced nucleoside uptake or activation, including loss of DCK for decitabine or UCK2 for azacitidine, can confer resistance to hypomethylating agents, while altered cytidine deaminase activity can reduce exposure (Schiffer et al., 2021). IDH-inhibitor resistance can emerge through secondary target-site mutations or isoform switching, and chromatin-directed therapies may be bypassed through compensatory recruitment of alternative transcriptional or chromatin-modifying complexes. Collectively, these mechanisms help explain why promising preclinical activity does not always translate into durable clinical benefit.

Despite these challenges, pharmacoepigenetics is increasingly moving toward biomarker-defined therapy, longitudinal epigenomic monitoring, rational drug combinations, and locus-specific editing. Integration of genomic and epigenomic data may improve prediction of efficacy and toxicity, while liquid-biopsy and single-cell approaches may capture evolving resistance states. Precision epigenetic editing offers the possibility of modifying disease-relevant transcription without permanent sequence alteration, and the clinical development of HBV-directed epigenetic silencing illustrates how this concept may extend beyond oncology. Continued development of validated predictive biomarkers and prospective trials linking epigenetic signatures to treatment outcomes will be essential for converting these mechanistic advances into individualized therapeutic decisions.

Although significant challenges remain, ongoing advances in epigenetic drug development, biomarker-guided patient selection, precision targeting using CRISPR based epigenetic editing and rational combination therapies are likely to expand the clinical utility of pharmacoepigenetics in oncology and other diseases including neurodegenerative diseases. Continued integration of genomics and epigenomic profiling into therapeutic decision-making may ultimately enable more individualized and durable treatment strategies.
